# Geomorphological assessment of the preservation of archaeological tell sites

**DOI:** 10.1038/s41598-023-34490-4

**Published:** 2023-05-18

**Authors:** Luca Forti, Filippo Brandolini, Valentina Oselini, Luca Peyronel, Andrea Pezzotta, Agnese Vacca, Andrea Zerboni

**Affiliations:** 1 Dipartimento di Scienze della Terra “A. Desio”, Università degli Studi di Milano, via L. Mangiagalli 34, 20133 Milano, Italy; 2 Istituto di Geoscienze e Georisorse, Consiglio Nazionale delle Ricerche, Via G. Moruzzi 1, 56124 Pisa, Italy; 3 McCord Centre for Landscape, School of History, Classics and Archaeology, Newcastle University, Armstrong Building, Newcastle Upon Tyne, NE17RU UK; 4 Department of Civil, Chemical, Environmental, and Materials Engineering, Alma Mater Studiorum University of Bologna, viale Risorgimento 2, 40136 Bologna, Italy; 5 Dipartimento di Studi Letterari, Filologici e Linguistici, Università degli Studi di Milano, via Festa del Perdono 3, 20122 Milano, Italy

**Keywords:** Geomorphology, Archaeology

## Abstract

Tells are multi-layered, archaeological mounds representing anthropogenic landforms common in arid regions. In such contexts, the preservation of the archaeological record is mined by ongoing climate changes, shift in land use, and intense human overgrazing. Such natural and human-driven factors tune the response of archaeological soils and sediments to erosion. Geomorphology offers a plethora of tools for mapping natural and anthropogenic landforms and evaluating their response to unremitting weathering, erosional and depositional processes. Here, we present a geomorphological investigation on two anthropogenic mounds in the Kurdistan Region of Iraq, with a special focus on the ongoing erosional processes mining their slope stability and threatening the preservation of the local archaeological landscape. Applying the revised universal soil loss equation model for soil loess derived from UAV imagery and implemented with geoarchaeological investigation, we assess the erosion rate along anthropogenic mounds and estimate the risk of losing archaeological deposits. We argue that a large-scale application of our approach in arid and semi-arid regions may improve our ability to (i) estimate the rate of soil and/or archaeological sediments loss, (ii) propose mitigation strategies to prevent the dismantling of the archaeological record, and (iii) schedule archaeological operations in areas of moderate to extreme erosion risk.

## Introduction

The formation, evolution, and preservation of archaeological landscapes and sites are ruled out by the interaction between natural surface processes of weathering, erosion and sedimentation, and human agency^1^. The latter include the ability of human groups of exploiting natural resources, coping with climate and environmental changes, and actively modifying natural landscapes, for instance modulating the intensity of surface processes, promoting the onset of anthropogenic geomorphological processes, building anthropogenic landforms^1^. Looking at the Holocene, the evolution of cultural landscapes lasted for several millennia, and the result is a palimpsest of natural and human-related landforms and deposits that formed under different environmental settings and as a response to changes in settlement types, land use, and subsistence strategies^2–5^ and deeply modified pristine environments.

Today, some of the processes—natural and artificial—in charge of the formation of archaeological landscapes and anthropogenic landforms may have been changed and new ones are involved in the dismantling or obscuring of the archaeological record (from site to landscape) due to erosion, over sedimentation, bioturbation (especially considering human as active agent), and intentional removal^2,6^. Such processes greatly threaten the preservation of archaeological sites (and in general of the cultural heritage) and hamper our ability in investigating past cultural dynamics. Geomorphology and geoarchaeology offer specific tools to explore archaeological sites and anthropogenic landforms and distinguish the origin of formative processes, evaluate the extant state of preservation of heritage, and thus plan scientific investigation. Moreover, such approach also allows to identify potential geomorphological risks and propose strategies for risk mitigation. Among others, soil erosion is one of the most significant environmental threats for the conservation of landforms as much as archaeological sites and monuments, especially in arid and semi-arid regions^7–11^. Recently, several models have been developed to estimate the rate of soil erosion, and among the others the Revised Universal Soil Loss Equation (RUSLE) has become the most adopted in a variety of environmental settings and at varying scales. The same model can be applied to archaeological soils and human-made landforms^10,12^, particularly in examining the impact of past land use practices on soil erosion rates^13^. Additionally, the RUSLE model can be employed to evaluate the effects of modern land use practices on archaeological landscapes^14–16^. Archaeologists can apply this method in different environmental settings the latter enabling them to predict the potential damage caused by erosion triggered by such activities and planning mitigation strategies for preserving the cultural heritage^72^.

In this paper, we propose a procedure for the geomorphological assessment of the potential risk for soil erosion on cultural heritage based on the investigation on two multiperiod tell-sites (tell means mound, sensu 7) in the Kurdistan Region of Iraq (KRI)—Tell Helawa and Tell Aliawa, whose stratigraphy spans from the pre-Halaf phase (ca. 7000 BCE) to the Early Islamic period (VII cent. AD)^17,18^. We carried out detailed archaeological and geomorphological survey coupled with the use of high-resolution UAV images and derived photogrammetric 3D models of each site to elaborate a digital surface model (DSM) employed to elaborate a RUSLE model for the two sites. This approach allows us to assess the rate of ongoing soil/sediment erosion affecting anthropogenic mounds, to reconstruct the evolution of the two tells, and identifying the major natural and human-driven ongoing processes threatening their preservation. We argue that the RUSLE model efficiently describes ongoing erosional processes along tell sites and offers a potential tool to identify geomorphological risks on archaeological sites. Moreover, this approach can be replicated at the regional scale, thus permitting to plan mitigation strategies to preserve endangered cultural heritage.

### The study area and the two sites

**Figure 1:**
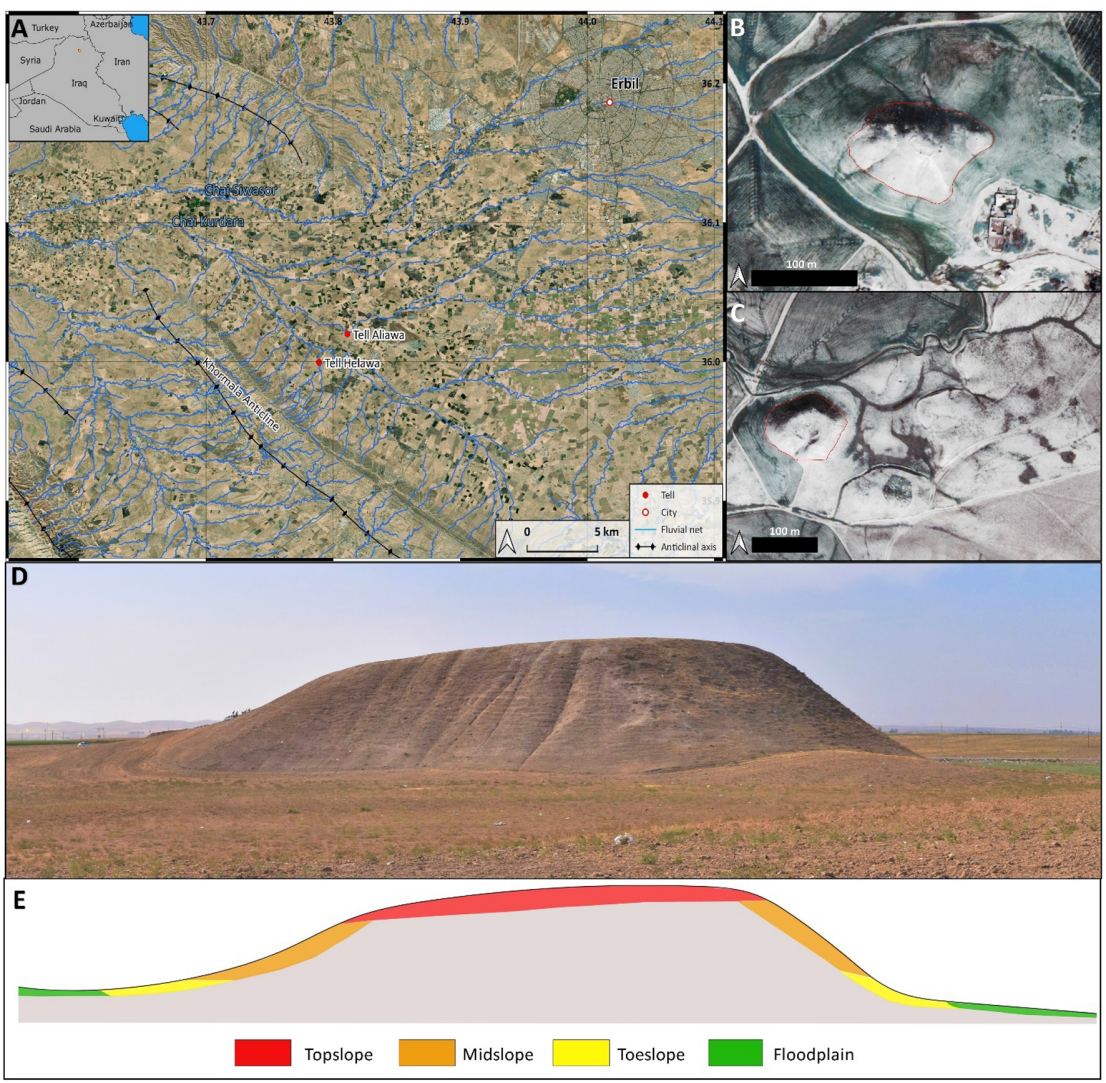
(**A**) GoogleEarth™ satellite imagery of the southern Erbil Plain crossed by a complex hydrographic network. (**B**) World View Imagery (12 March 2011) of Tell Helawa (red line indicates the mounds). (**C**) World View Imagery (12 March 2011) of Aliawa multiperiod mounds (red line indicates the main mound) (elaborated with QGIS 3.16.7 plugin https://nextgis.com/blog/quickmapservices/). (**D**–**E**) Theoretical model not to scale (based on the main mound of Aliawa) illustrating the different topographic areas of a tell as discussed in the text. (Archive of the MAIPE Archaeological Project of the University of Milan) (Elaboration maps, picture, and artwork L. Forti).

The study area is in the Kurdistan Region of Iraq (KRI) within the Erbil Governorate (Fig. 1A), along the Foothills Zone of the morphotectonic region related to the structuration of the Zagros orogen^19–23^. Helawa and Aliawa are two multi-layered anthropogenic mounds located ca 28 km SW of Erbil, at the foothills of the Khormala Anticline (Fig. 1). The tells arise along a wide flat flood plain, characterized by a reddish silty clay to clay deposit, corresponding to the Chai Kurdara (meaning Kurdara River) alluvial plain, which is a left tributary of the Great Zab River. The plain is dissected by two SE–NE-oriented tributaries of the Chai Kurdara and today exploited for agriculture and grazing purposes (Fig. 1B–C). Climatic data from the computational models available for Erbil suggest a semi-arid to temperate warm climate with an average annual rainfall around 400 mm/y and temperatures ranging from − 2 to 38 °C. Winters are wet and cold, concentrating the 90% of the annual rainfall between December and March; summers are dry and warm to extremely hot^24,25^. Few paleoclimatic data are available to reconstruct local Late Quaternary environmental changes. Most of data are derived from stable isotopes analysis on speleothems and lake deposits and recorded several climate fluctuations happened after the Last Glacial Maximum. Hydroclimatic proxies from the Eastern Mediterranean recorded a shift from the cold and dried condition of the Younger Dryas (13–11.7 ka BP) towards warmer/wetter conditions in Early Holocene (11.7–8.5 ka BP), followed by a progressive increase of aridity in the Middle and Late Holocene^26–32^. Increased aridity potentially increased soil degradation and enhanced soil erosion^33^.

In south-western Asia especially, tell-sites are one of the major anthropogenic features rising from the surrounding flat area and representing an example of Holocene anthropogenic landforms. Multi-period mounds are confined raised accumulations of multiple archaeological layers of building levels and wastes, which grew up through time due to stationary occupation of the site and subsequent phases of in-situ buildings decay^1,7,34^. The Erbil Plain is a densely settled and human-modified landscape with hundreds of archaeological sites, including tells, identified by means of extensive survey coverage carried out by the EPAS Expedition of the University of Harvard over the last decade^35,36^. Targeted ongoing excavations at different sites are also providing a reliable chronological scheme for the reconstruction of human occupation and landscape exploitation through time^35,37,38^. Among the others, the multi-period mounds of Helawa and Aliawa are noteworthy anthropogenic features, investigated since 2013 by the MAIPE (Missione Archeologica Italiana nella Piana di Erbil) Archaeological Project of the University of Milan. The two sites together allow to reconstruct a long occupational sequence spanning from the prehistoric period to the Islamic age^17^. In fact, Helawa (Fig. 1B) shows a mainly pre- and proto-historic archaeological sequence dating from the pre-Halaf to the Late Chalcolithic 3 (∼ 7000–3600 BCE), followed by a period of abandonment and a short-term re-occupation during the early Late Bronze Age (∼ 1550–1400 BCE). The site of Aliawa (Fig. 1C) is instead probably occupied during the Ubaid period (∼ 5300–4500 BCE) and extensively settled during the Early to Late Bronze Age (∼ 3000–1200 BCE), as well as from the Iron Age (∼ 1200–550 BCE) until the Hellenistic/Seleucid and Parthian periods (late fourth-century BCE–second century CE) with the latest settlement dated to the Late Islamic period.

## Methods

### Remote sensing and geomorphological mapping

**Figure 2:**
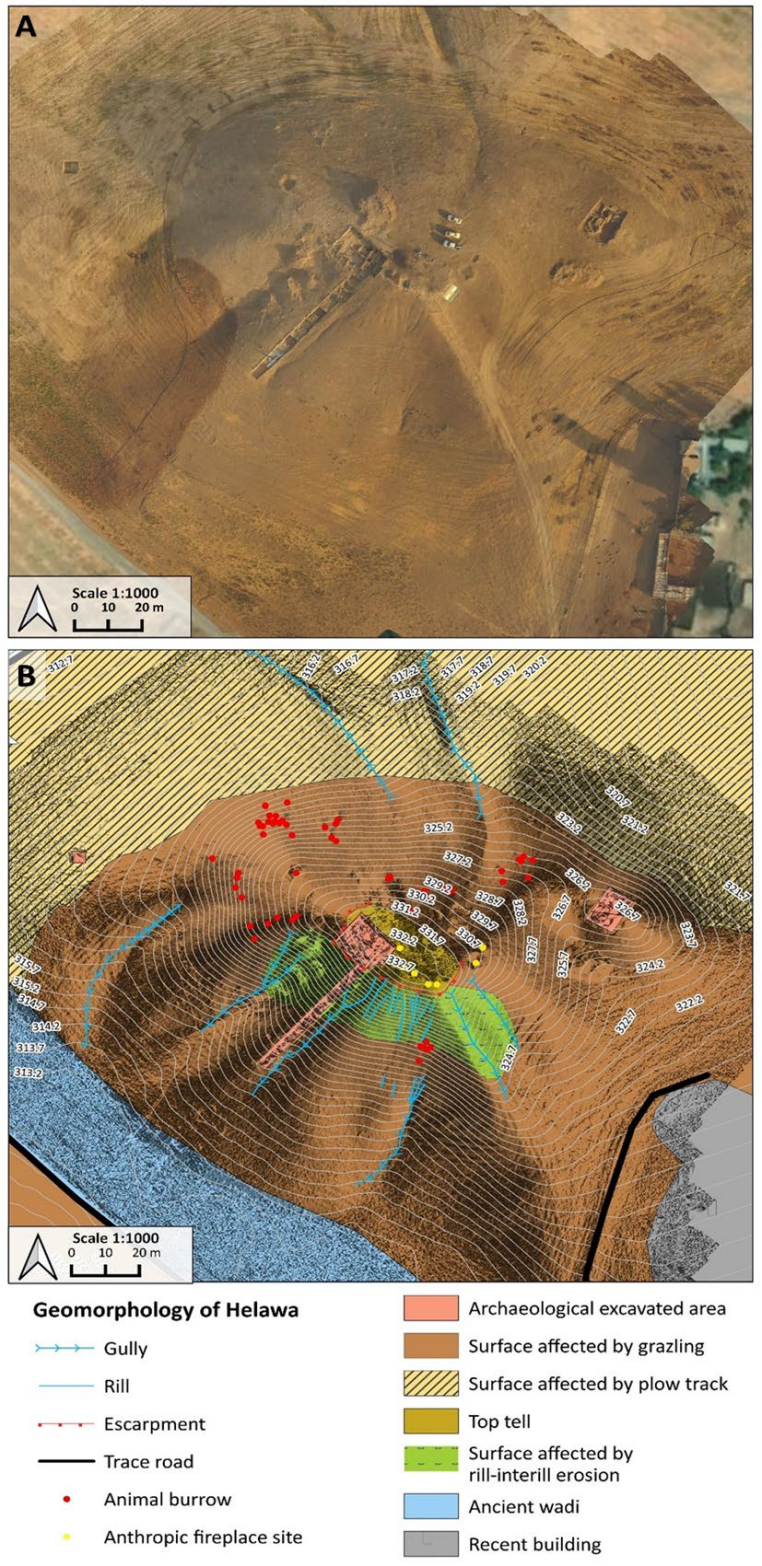
Geomorphological mapping of Helawa. (**A**) Orthophoto of the tell elaborated from the UAV pictures. (**B**) Detailed geomorphological map of the tell illustrating the main landforms and active processes; Archive of the MAIPE Archaeological Project of the University of Milan). (Maps elaboration L. Forti).

High resolution geomorphological and archaeological mapping of Helawa and Aliawa has been performed both on the field and from remote sensing^18,37^. In fall 2021, we acquired aerial pictures of the two areas performing a detailed UAV fly at 30 m above the mounds; additionally, several nadiral photos of the sites and their surroundings were taken to obtain a detailed and updated topography of the two sites. High resolution 3D digital models of archaeological sites and features are commonly used in geo-archaeological research and elaborated starting from ground-acquired information (e.g., with laser scanner) and airborne data, as the LiDAR ones^39–41^. Lacking such facilities, as in the study region, the application of photogrammetry based on the use of small and low-cost UAVs equipped with commercial cameras is becoming common practice^42^, and the same methods are increasingly adopted in geomorphology^43^. This approach permits to gather high-definition pictures useful to elaborate photogrammetric models of archaeological sites^44,45^. In archaeology, such reconstructions represent a tool to assess the shape and extension of sites and to measure the surface distribution of archaeological materials and features^46,47^. Yet, only occasionally photogrammetric models have been applied to assess past and ongoing geomorphological processes affecting the archaeological record^48–51^. In our case, the high-resolution mapping of the two tells was performed using an UAV DJI Phantom 4 with a flight at 30 m above the mounds and several nadiral photos taken to obtain a detailed and updated topography of the two sites. More than 149 photos were taken for the site of Helawa and 235 for Aliawa to achieve a 60% overlap, they were taken in regular parallel movements to reduce data loss. The images are at a 72-dpi resolution with 12,000 pixels. Oblique aerial photos were imported and processed into Agisoft Metashape Professional (Version 1.5.5)^52^ with the standard workflow that includes photo alignment, built of dense cloud and mesh to produce a 3D model of the two sites with the extrapolation of Digital Surface Model (DSM). Afterwards, DSMs were imported in QGIS 3.16.7^53^, and a hillshade model with contour lines at 1 and 0.5 m and a classification of streams were generated. High resolution geomorphological and archaeological mapping of Helawa and Aliawa has been performed both on the field and from remote sensing^54^, based on the observation of WorldView2 (acquired 12 March 2011) and Google Satellite Imagery for basemap visualized through “QuickMapServices” plugin on QGIS 3.16.7^55^. For sake of clarity, a topographic theoretical model of tell topography is represented in Fig. 2, reporting on the major parts of a tell and explaining the terminology here adopted: the uppermost part of a tell is the topslope, its middle part is the midslope, and the lowermost sector of the mound, connecting the tell with the surrounding floodplain, is the toeslope (Fig. 1D–E).

### Soil erosion modelling

To estimate long to term average annual soil loss and to map erosion hazard at Helawa and Aliawa a RUSLE empirical model was developed. RUSLE’s elaboration requires five different factors describing the environmental settings of the region of interest and is based on the Eq. (1):

1 $$A = R \times K \times LS \times C \times P$$

where R is the rainfall erosivity, K the soil erodibility, LS is the slope-length topographic factor, C and P represents the land cover and land management variables respectively, and A is the resulting average annual erosion rate measured in tonnes/hectare/year. Several methods have been developed in the last decades to calculate the five RUSLE factors^56^.

The R factor [MJ mm ha^−1^ h^−1^ year^−1^] represents the impact of rainfall that causes soil erosion^57^, and in this research, it was defined according to the formula (2) proposed by^58,59^:

2 $$R = 0.048380*P\;^{ \wedge } 1.610$$

where P is the mean annual precipitation rate. This method has been demonstrated particularly effective in areas where *P* < 850 mm/year^13,14^. According to CRU TS (Climatic Research Unit gridded Time Series) dataset elaborated Harris et al.^24^ in the study area, the average rainfall is 419.45 mm/year therefore the resulting R factor is 807.556536.

The definition of the Soil Erodibility Factor (K) is derived by the geomorphological processes behind the development of the two tells considered. As suggested by Menze and Ur^60^, tell sites consist of anthrosols, meaning soils that have been deeply modified by human activities^61^. In literature, the corresponding K factor value for anthrosols is 0.30^62^. The topographic factor LS can be divided into two separated factors: the slope length L-factor and slope steepness S-factor. The DSM generated with a photogrammetric approach has been employed to calculate the LS factor in GRASS GIS^63^ with the *r.uslek* module. The values for the land cover and crop management factor (C) were collected from the literature (see Table 1), while the support practice factor *P* is not considered (i.e., *P* = 1) because erosion control measures have never been established in the study area. The resulting RUSLE models were reclassified into five erosional risk categories: minimal (0–10 tonnes/hectare/year), very low (10–30), low (30–60), moderate (60–120), severe (120–150), and extreme (> 150).

**Table 1:** C Factor values considered in this study.

Land cover type	C Value	References
Fair Graziland	0.16	^64^
Poor Graziland	0.30	^64^
Continuos Cropland	0.50	^64^
Natural Grassland	0.0435	^65^

## Results: Geomorphological assessment of ongoing processes at tell sites

### Helawa

The site of Helawa is located near a small village, on the right bank of a watercourse characterized by a low sinuosity that flows around the site and merges into one of the tributaries of Chai Kordara (Fig. 1B). The site is a subrounded mound composed of two heights, the highest rising ∼ 22 m above the floodplain level (top at 332.7 m a.s.l.), and the lowest, located to the north-east of the main one, rising ∼ 16 m above the floodplain (top at 326.7 m a.s.l.) (Fig. 2A)^17^. The Helawa mound lays along a bend of an ephemeral stream surrounding the tell at the foot of its western and south-western slopes.

**Figure 3:**
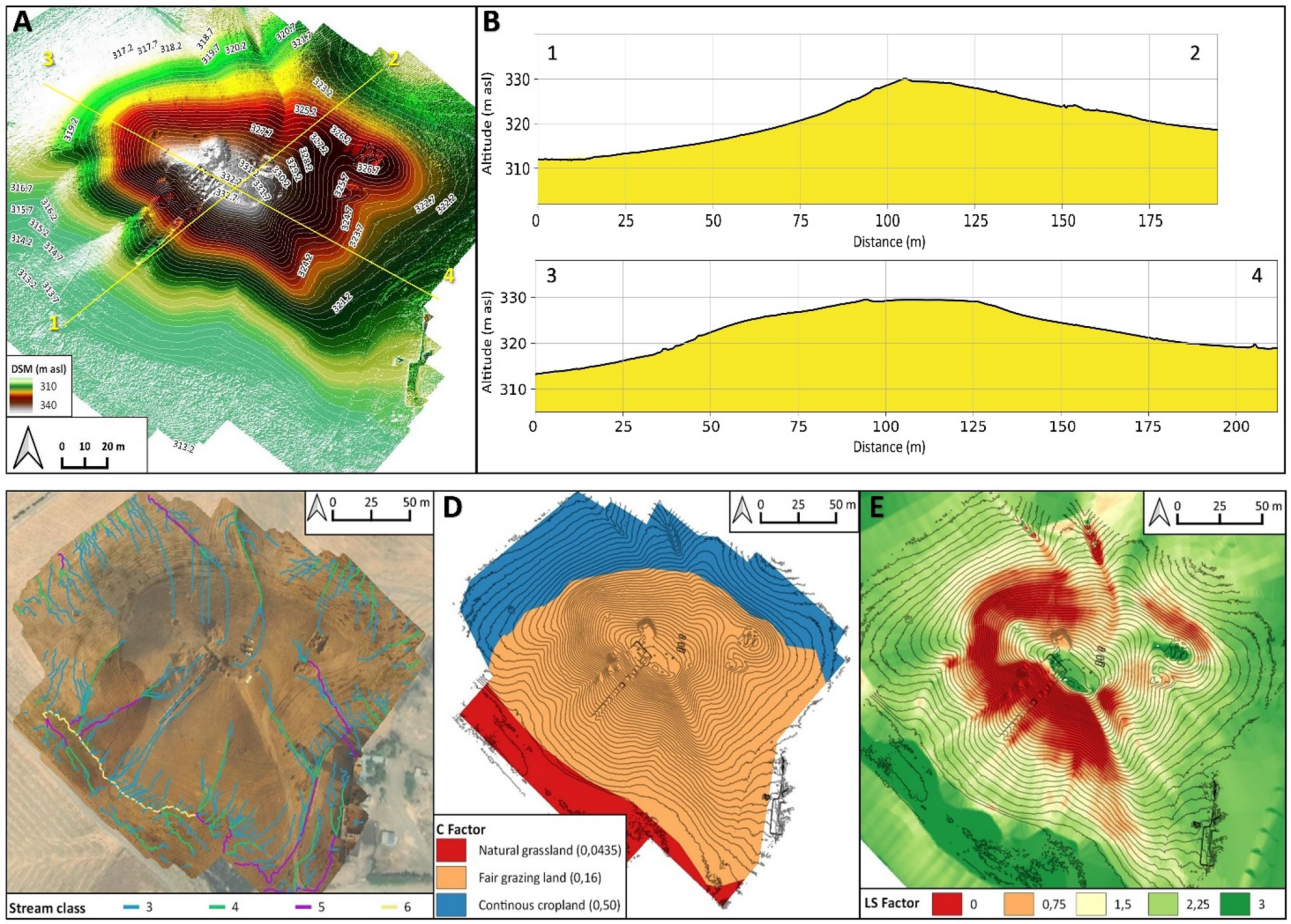
DSM and data required for the elaboration of the RUSLE model of soil erosion for Helawa. (**A**) DSM of the tell elaborated from the UAV pictures; the position of cross sections of (**B**) are reported. (**C**) The drainage network of Tell Helawa extrapolate from the DSM analysis. (**D**) C and (**E**) LS factors elaborated with QGIS software (Archive of the MAIPE Archaeological Project of the University of Milan) (Maps elaboration L. Forti).

**Figure 4:**
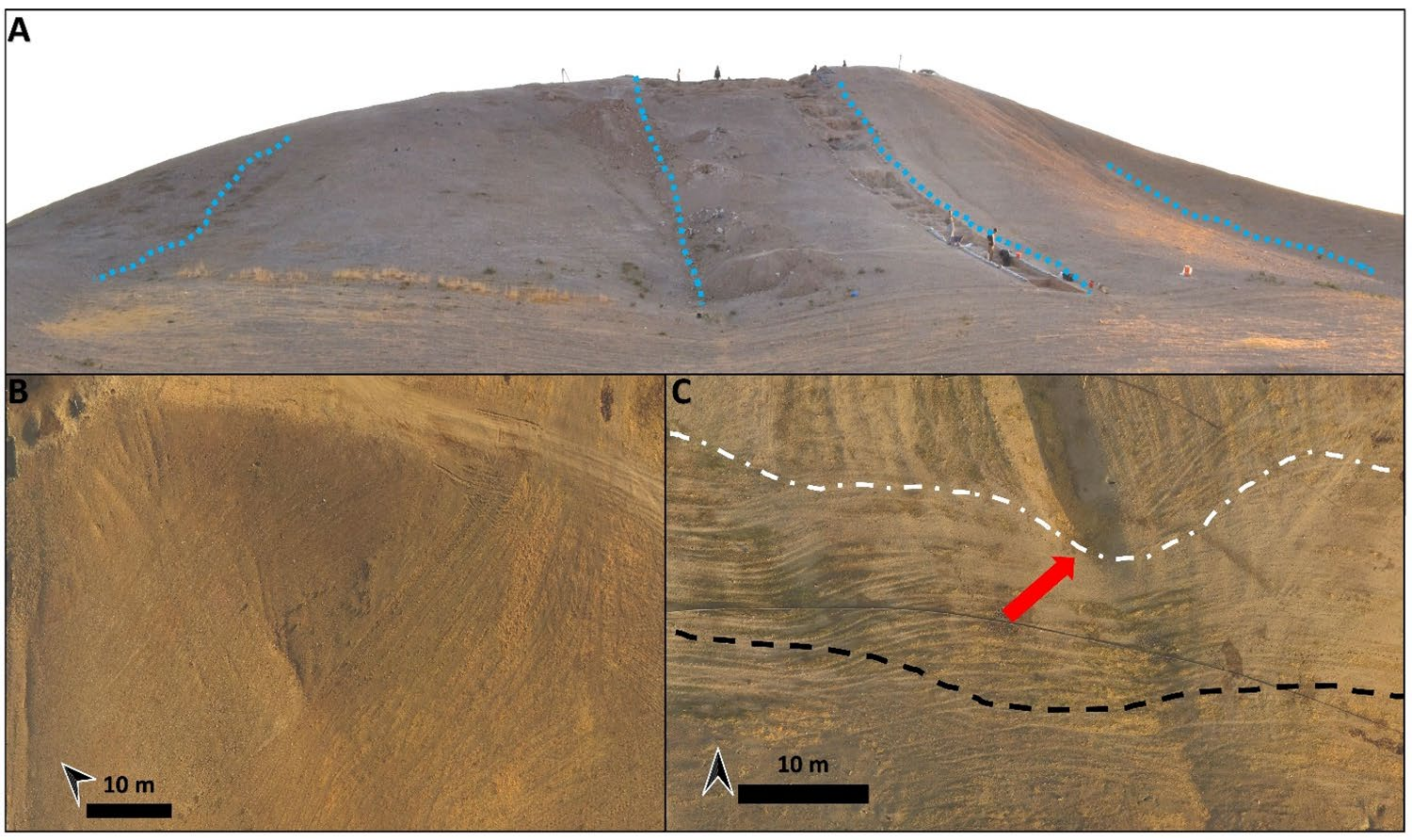
Field pictures of Helawa. (**A**) Southern side of the tell, where main gullies (indicated by the blue dashed lines) deeply cut the slope of the mound. (**B**) UAV imagery illustrating the southern side of Helawa that is crossed by a rill-interill network. (**C**) UAV imagery of the northern toeslope highlighting the transition between the toeslope (white dashed line) and grazing and this latter with cropping belts (black dashed line); this transition is marked by the increased depth of gully in correspondence of the plough track furrows (indicated by the arrow). (Archive of the MAIPE Archaeological Project of the University of Milan) (Pictures and artwork L. Forti).

The geomorphological survey highlights that the N and NE sectors of Helawa are nowadays occupied by cultivated fields, whereas the effects of continuous livestock trampling (pathways) is evident on the western side of the tell (Fig. 2B). Channels related to rill-interill and gully erosion are evident along the slopes. Gullies are natural, but occasionally their development is supported by human intervention; a further control over the formation of gullies is played by the topography of several areas of the mound, and specifically by lines of weakness related to the assessment of the mound after subsequent phases of human occupation. Therein, rill-interill and gullies as well as processes related to human and animal agency are the main factors mining the preservation of the archaeological record (Fig. 2B). The topographic profile extracted from the DSM (Fig. 3A) highlights how slopes have two different shapes based on the topographic gradient. Two sections, one oriented W–E (profile 1–2 in Fig. 3) and the second oriented NW–SE (profile 3–4 in Fig. 3), show that the southern and south-western sectors of the mound are steeper than the northern and eastern ones and the top of tell is almost flat. The uneven setting of the mound slopes has consequences on the response to ongoing surface processes, thus differentiating the impact of erosion. In fact, along the S–SW edge of Helawa the rill-interill network carved 4 major gullies, that show an increasing rate of incision from upstream to downstream that is likely mitigated by the decreasing slope gradient (Figs. 3C, 4A–B). Two major incised gullies, flowing from topslope northwards, are distributed along the gently northern slope. These gullies display a confinement setting with an increased rate of incision downstream that is highest at the toeslope, where the topographic gradient changes. Besides the obvious control of slope gradient, gullies’ hydrodynamics along the tell slope is additionally driven by the different land use of each patch of ground: we notice that the shift from herding-related trampling to ploughing modify the rate of incision from the topslope to the toeslope (Fig. 4C). The western toeslope is gently connected to the right bank of the low sinuosity stream and displays a sparse grass cover and domestic livestock trackways (Fig. S1A).

The evaluation of the erosional risk along the tell, based on the analysis of the DSM reveals that only 4 out of 6 identified stream classes (namely, class 3–6) are active after heavy rainfall; stream classes develop along the topslopes, toeslopes, and surrounding crop fields. The third and fourth stream classes encompass the shallow rill-interill network, while the fifth and sixth classes include the deep gullies and the uppermost reaches of the stream network (Fig. 3C). Stream classes are useful for categorizing streams based on their size, shape, and surrounding landscape. In the context of erosion of archaeological sites, stream classes are relevant because they can help in understanding the potential for erosion to occur in each area. For example, streams with a high stream class (i.e., larger and more powerful streams) are more likely to increase the rate of erosion than streams belonging to a low stream class (i.e., smaller, lower streams). The land cover (C), indeed, is categorized into 3 different classes: the first is grassland that is partly affected by the grazing of livestock, the second and third classes are the areas deeply affected by grazing and cropping activities (Fig. 3D). The ratio between length and slope gradient (LS) displays that the maximum values are in the in southern and northern midslopes and in the proximity of gullies incision, while the minimum are in topslope, toeslope, and in the surrounding floodplain (Fig. 3E). Along the northern and southern midslope, the maximum values of LS can be explained considering anthropogenic factors contributing to the shaping the mound, as for instance the substantial levelling, terracing, and building activities occurred during the Ubaid and Late Chalcolithic periods^18^. In fact, archaeological excavations carried out along the southern slope of the mound in Step Trench B (over a N–S total length of 70 m and an E–W width of 4 m) allowed documenting a packed sequence of monumental buildings and the generalised use of high, and in some cases relatively narrow, terraces during the Ubaid and Late Chalcolithic 1-3 17. Such terracing activities greatly contributed driving the actual shape of the tell and modulating the topographic control over erosion.

### Aliawa

**Figure 5:**
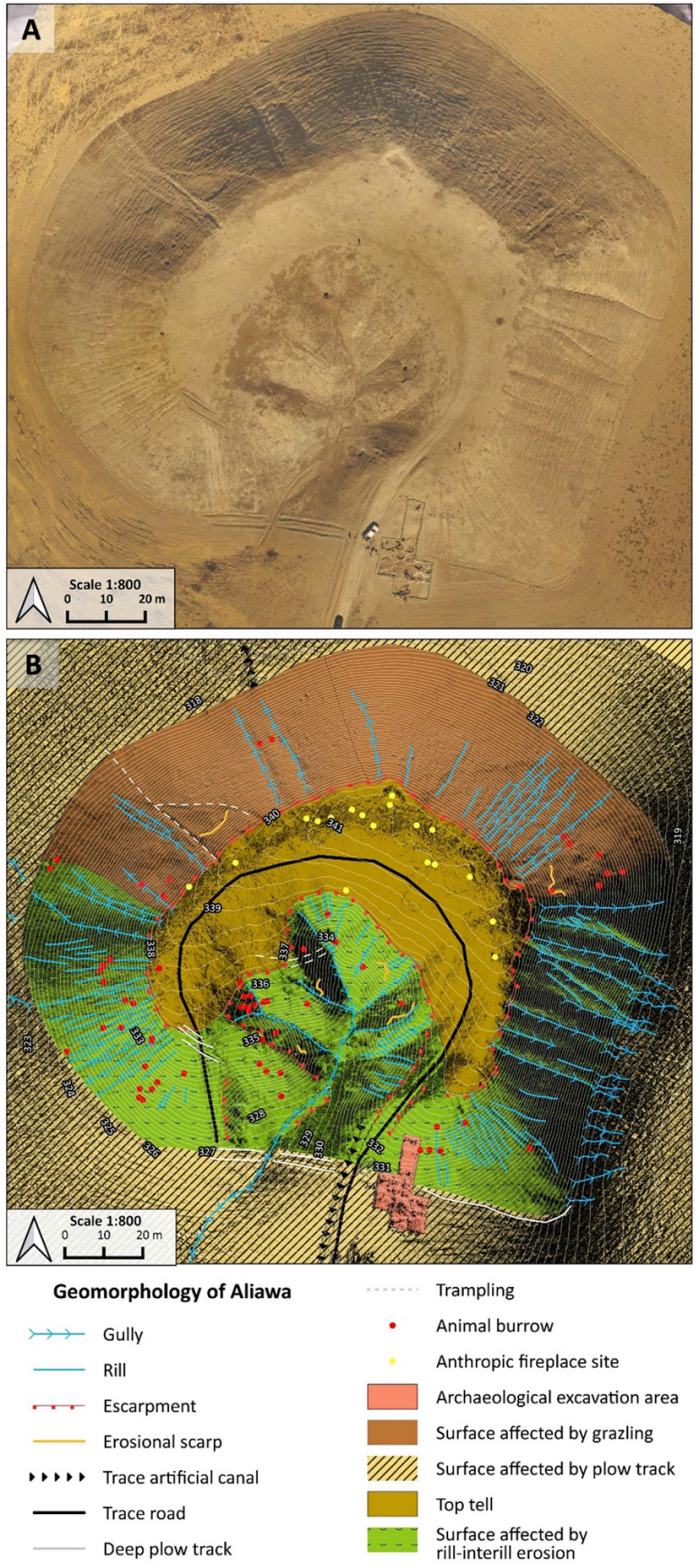
Geomorphological mapping of Aliawa. (**A**) Orthophoto of the mound elaborated from the UAV pictures. (**B**) Detailed geomorphological map of the tell illustrating the main landforms and active processes (Archive of the MAIPE Archaeological Project of the University of Milan) (Maps elaboration L. Forti).

The general morphology of Aliawa is more articulated than that of Helawa. The site extends over a surface of ∼ 250.000 m^2^ and consists of a main mound rising 23 m above the floodplain (top at 341 m a.s.l.) and ∼ 25.000 m^2^ large. The main mound is flat-topped and surrounded by several lower mounds (rising 3–5 m above the floodplain), flanked by an ancient watercourse that runs along the northern edge of the site (Fig. 1). In this work, we only consider the processes acting along the main mound, which therein is indicated as Aliawa (Fig. 1). The archaeological record suggests that the prehistoric and Bronze Age shape of Aliawa was modified by the construction of a high fortress bordered by brick embankments, probably dating to the Hellenistic/Seleucid periods. Such chronological attribution is preliminary and needs further excavation to be verified. In any case, a pentagonal perimetral wall was built over the mounds resulting after preclassic occupation (∼ 3000–1200 BCE)^37^. As a result, the extant steep slopes of the tell are the perimetral defences of the later fortified settlement, whilst the gently slope below the wall is part of the earlier Bronze Age settlements; the latter is especially visible in the southern sector of the mound, where it was not covered by the fortress. Today, the top of Aliawa is flat, while its central part is a recessed area, interpreted as an inner space opened behind the fortress gate and then affected by intense natural and anthropic erosion (Fig. 5A).

**Figure 6:**
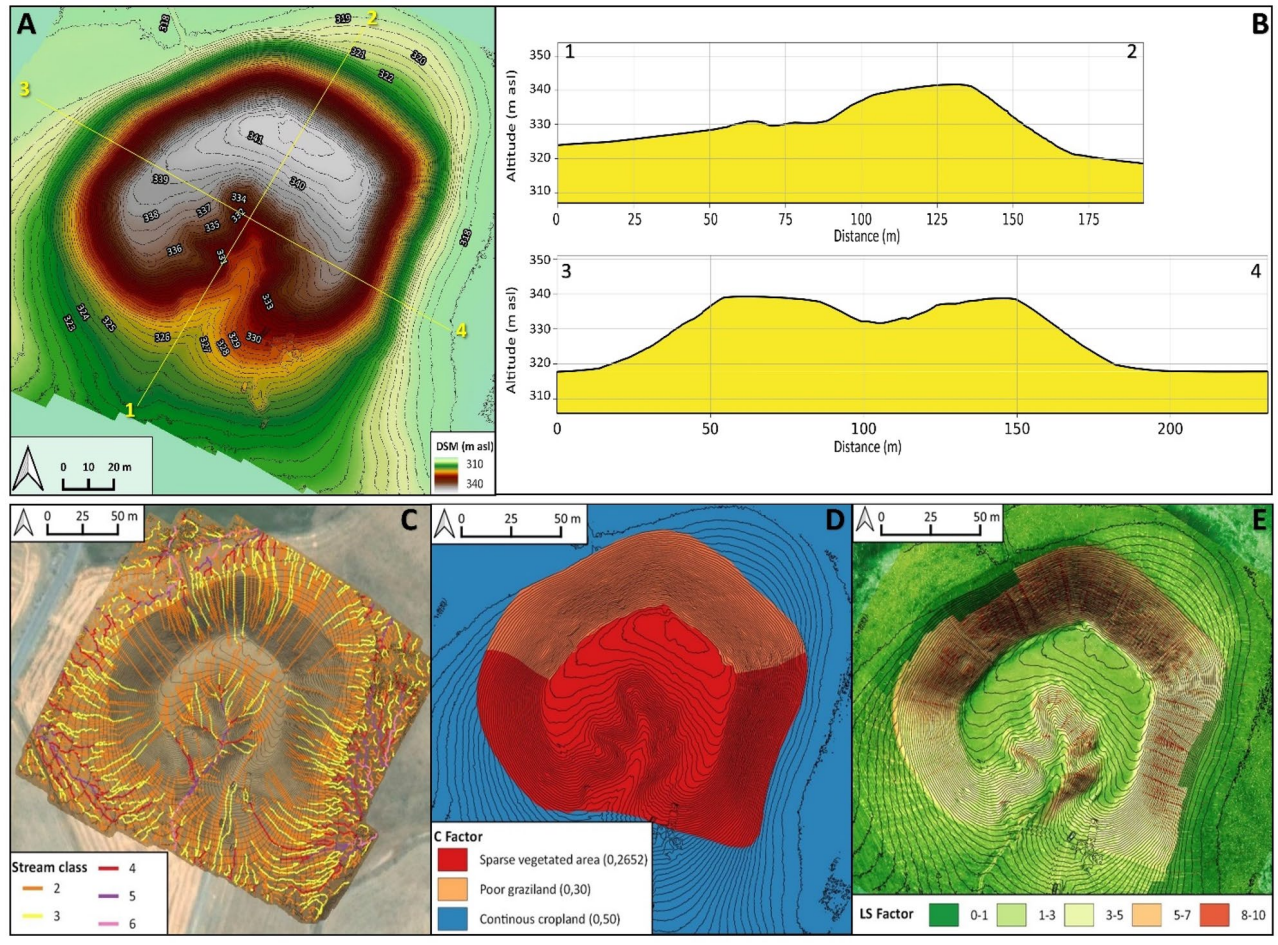
DSM and data required for the elaboration of the RUSLE model of soil erosion for Aliawa. (**A**) DSM of Tell Aliawa elaborated from the UAV pictures indicating the position of cross sections of (**B**), that are illustrated in the text. (**C**) The drainage network of Aliawa extrapolate from the DSM analysis. (**D**) C and (**D**) LS factors elaborated with QGIS software (Archive of the MAIPE Archaeological Project of the University of Milan) (Maps elaboration L. Forti).

**Figure 7:**
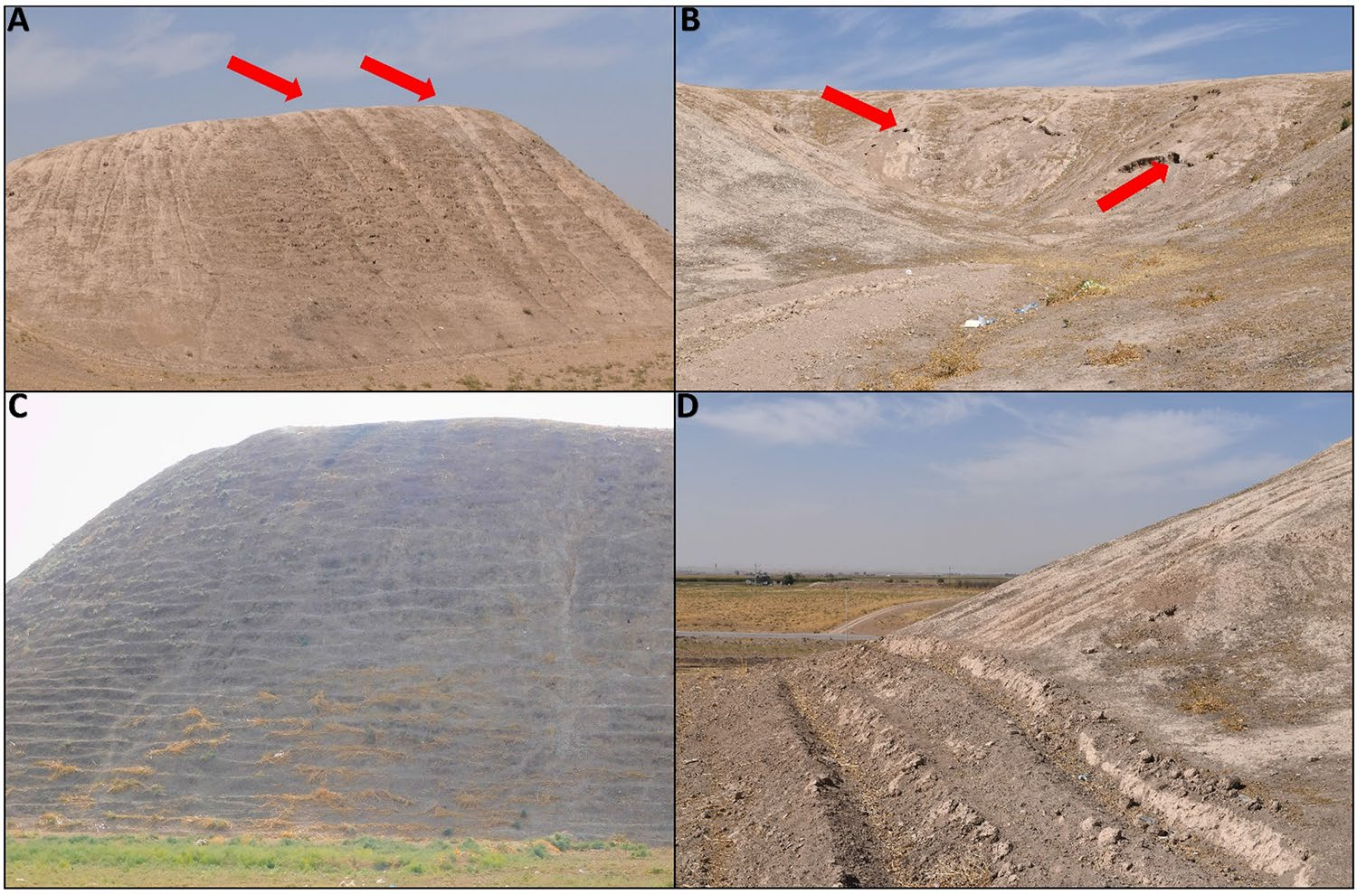
Field pictures on the main erosional features affecting the slopes of Aliawa. (**A**) Linear interill-rill network along the eastern slope of the tell (indicated by arrows). (**B**) The badland-like basin in the central part of the tell, developed in the internal part of the Seleucid fortress after the interplay between the rill-gullies erosion and the animal burrows (red arrows). (**C**) Sheep tracks following the direction of contour lines along the northern slope of the tell (sheep tracks follows the alignments of bricks of the Seleucid wall). (**D**) Deep plough furrows at the toeslope of the tell along the southern gentle slope (Archive of the MAIPE Archaeological Project of the University of Milan) (Pictures and artwork L. Forti).

Detailed geomorphological mapping (Fig. 5B) shows that the main mound suffered erosional processes, including the rill-interill network evident at the southern side and in the central part of the tell, a well-developed track sheep along its northern slope, and ploughing at its toeslope. Again, the slope gradient modulates the intensity of ongoing geomorphological processes; this is suggested by the analyses of two topographic profiles extracted from the DSM (Fig. 6A). The SSW–NNE profile (section 1–2 in Fig. 6A) displays a southern gentle slope with a progressive increase of the topographic gradient toward the S, up to the top of the tell. Yet, the northern slope shows a steep gradient with an abrupt transition to the surrounding flat area (Fig. 6B). The E–W profile (section 3–4 in Fig. 6) highlights the steepness of the eastern and western slopes of the mound (related to the fortress walls) and the central depression (Fig. S1B). Topographic profiles underline the different slope gradient of the tell, with a gently slope in the southern part (Fig. S1B) that becomes steeper toward the N (Fig. S1C) and in the eastern and western sectors. Downstream the rill-interill network, the rate of incision increases and at the toeslope of the mound and the overland flow is drained into a gullies system developed along ploughing furrows (Fig. 7A). Our survey suggests that the central part of the main mound is a sort of small badland-like basin, whose formation follows the excavation of the recessed area of the fortress. Therein, the original shape of the recessed area is deeply modified by the coalescence of several rills-gullies branches (Fig. 7B), and several collapse scarps triggered by excavation of animal borrows (Fig. 7B) and looting holes. The main features recorded along the northern mid-slope corresponding to the fort walls are several trails that runs parallels to the slopes according to contour lines of rows walls referred to domesticates trampling (Fig. 7C). Downslope, the boundary between the tell and the surrounding plain is marked by a shift in land use from animal grazing to cultivation; again, this results in a network of shallow and deep ploughing furrows (Fig. 7D). The latter drain and redistribute the rainwater from the runoff of the downslope to the surrounding crop fields, as common in the contour farming practise^66^. Modern fire pits and trackways made by cars are further anthropogenic disturbances present on the top of Aliawa.

Modelling on DSM of Aliawa detects 5 different stream classes (from 2 to 6). In details, the second and third classes are the shallowest drain network, while from the depth of incisions of the fourth, fifth, and sixth stream classes increase along the inner part of the tell and along the toeslope (Fig. 6C). The land cover (C) of Aliawa is characterized by sparse grassland vegetation, especially in the southern sector, while the northern slope is affected by livestock trampling and the surrounding area of the tell by cultivation (Fig. 6D). The ratio between length and slope gradient (LS) highlights that the minimum values are in the flat area such as the top of the tell and the surrounding cropping fields, while the maximum are in the slopes and in the middle of the badland-like basin (Fig. 6E).

## Discussion on erosional processes affecting the preservation of the archaeological record

Since its origin, the landscape surrounding Helawa and Aliawa—including the anthropogenic landforms represented by the two mounds—is deeply influenced by the interplay between natural and biogeomorphological (human and animal-controlled) surface processes. At multiple scales of resolution, such agencies oversee soil erosion and strongly impact on the conservation and preservation of the archaeological record. This is evident considering the nature of tells; in fact, they are entirely composed of superimposed (and decaying) clay buildings, archaeological materials and architectonic structures thus representing landforms covered by anthrosols^60^. The local climate promotes natural erosion processes leading to the formation of the rill-interill and gullies drainage network, which deeply carve the slopes of the tells. Human and animal agency further fuels erosion, enhancing the effect of linear erosion and increasing the rate of soil loss^67–69^. The geomorphological mapping shows that erosional processes are especially severe along the slopes of tells, where goats/sheep’s trampling and the animals borrow trigger the mobilization, transport, dispersal, and secondary re-deposition of archaeological sediments and materials.

**Figure 8:**
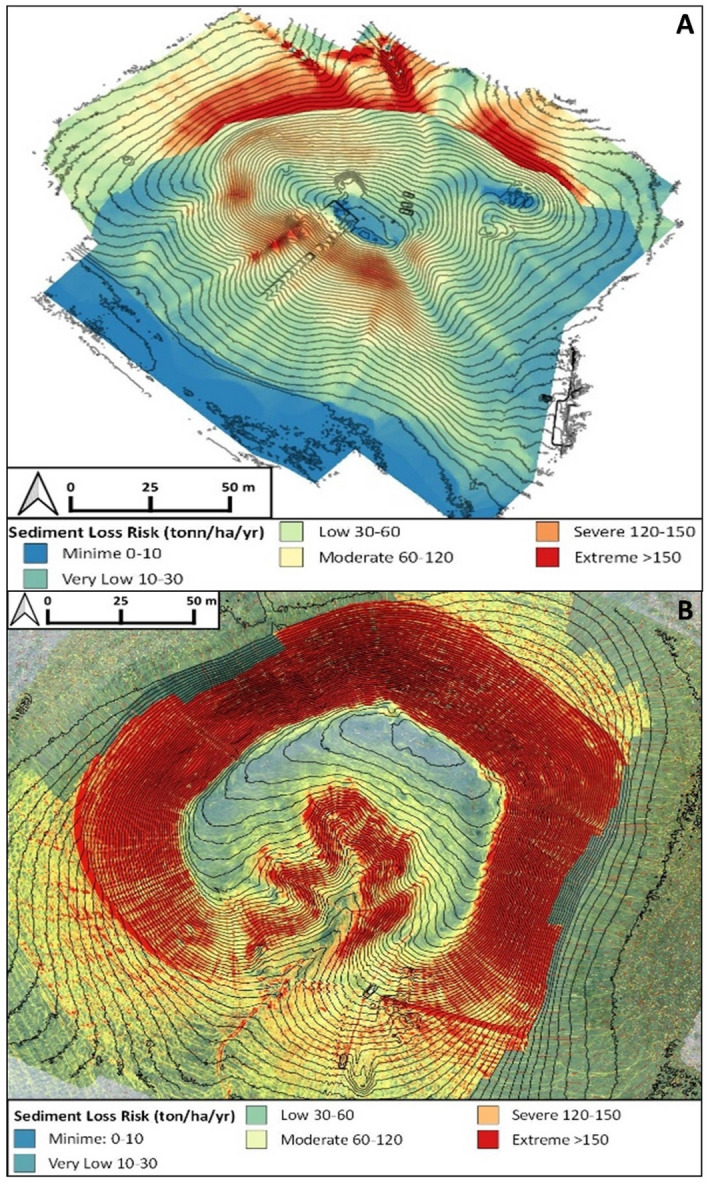
RUSLE models elaborated for (**A**) Helawa and (**B**) Aliawa. The colour scale (from blue to red) represents the increased risk for soil/sediment loss calculated in ton/ha/yr. The red areas are those where the archaeological record is mostly endangered by ongoing erosional processes (Archive of the MAIPE Archaeological Project of the University of Milan) (Elaboration maps L. Forti).

The RUSLE models elaborated based on the interpolation between data derived from land cover, land management, type of soil, and the local amount of rainfall, show different degrees of geomorphological risk induced by erosion at different parts of each tell sites (Fig. 8). The RUSLE models of Helawa and Aliawa also suggest that the tells’ topography and the gradient of slopes are the main factors that promote erosion and trigger the loss of soil and/or archaeological sediments. The susceptibility to soil loss increases where the slopes switch from moderate to steeply; such change in topography is often related to the occurrence of specific archaeological structures (e.g., terraces). Moreover, potential erosion increases at the toeslope of each site in correspondence of ploughing furrows and drain canals.

In the case of Helawa, the RUSLE model shows that the highest loss of soil and/or archaeological sediments occurs along the SW-NNW side of the mound (along the belt comprises between 325 and 330 m a.s.l.), and along the N-NE side of the mound at the transition between the toeslope and the floodplain. These two different high values of estimated soil loss can be interpreted in two different ways. Along the SW-NNW part of tell, high values of soil loss are likely imputed to the interplay between the intense construction activities occurred in the Late Chalcolithic, which made the current slope very to moderate steep; this favoured the inset of gullying^18^. Instead, along the northern side at the transition with the floodplain extreme to severe values of soil loss estimation are related to a decrease in slope gradient coupled to intense ploughing operations, resulting into an intense soil erosion due to gully formation. A moderate to low geomorphological risk for soil and/or archaeological sediments loss is suggested for the southern and western sides of Helawa and along its northern toeslopes, where grassland covers the surface, and the LS ratio is low (Fig. 8A). With respect to Aliawa, the RUSLE model highlights that the highest potential risk of soil and/or archaeological sediments loss is in the inner part of the tell and around the midslope. In such areas, erosion is fuelled by the rill-interill network, animal burrowing, and the contour tracks sheep. At the same site, moderate to low values of erosion risk were detected along the cropping area and at the top of the settlement, where the slope gradient is very low (Fig. 8B). The distribution of soil loss estimation values at Aliawa is influenced by the shape derived from the destruction and obliteration levels of the original prehistoric and Bronze Age settlement due to build activities of fortress. Hence, moderate to low values of erosion risk are recognized at the northern toeslope and in the southern side of the tell; such parts correspond to the slope of the original settlement. Yet, moderate to severe erosion risk is suggested for the parts of the mound corresponding to the walls of fortress, and in the central part of the mound (the artificial recession).

In detail, the calculated potential soil loss elaborated from topographic parameters shows a lower susceptibility to erosion at Helawa than Aliawa. This is a snapshot of the current situation, resulting after centuries of erosion along the slope of tells and the building of fortification walls along the perimeter of Aliawa. If we consider field evidence, we notice that the toeslope of Helawa is composed of a large dispersion of archaeological sediment eroded from the Bronze and Late Chalcolithic layers of the tell and deposited in the area surrounding the site. This means that likely in the past, when the topography of the site was different, erosion rate was more intense than today. Yet, at Aliawa a few or clustered archaeological reworked sediments are dispersed along the toeslope of the tell, likely because the later budling of perimetral walls protected the pristine tell’s stratigraphy from runoff and erosion. We suggest that different re-distribution of archaeological sediments at the toeslopes of each site was mostly controlled by erosional processes occurred in the last centuries (after the Seleucid occupation of the area), likely after the transformation of Aliawa into a fortress.

This hypothesis highlights that the amount of archaeological sediment loss at Helawa and Aliawa is triggered by the time of exposure to erosional processes. For that reason, today Aliawa displays higher values of potential soil loss than Helawa, but it is a consequence of the modification of the tell shape occurred during recent occupational phases; in fact, the fortified walls modified the topography of the site and increased the slope gradient. Finally, along Aliawa rills are more common and shallower compared to Helawa, where rills are concentrated at the southern edge of the mound with gullies that deeply cut the southern and northern sides of the tell (Fig. 4A).

In conclusion, we noticed that the exposure to soil erosion values at each site is strongly influenced by the different shape of the settlements, depending on their evolution and human re-shaping. Helawa shows high values near the southern slope, where the different phases of the Chalcolithic construction have made the slope unstable and steep. This factor led to the consequent abandonment and later, Bronze Age, relocation of the settlement to the northern sector^18^. At Aliawa the pristine mound is preserved by the later fortifications, but the mound shows higher erosion risk values. This is due to the presence of the steam fortification walls and the opening of the inner recession, which exposed the archaeological stratigraphy to intense surface processes. Therefore, the different nature and archaeological history of each tell shaped the current morphology of mounds and drive the intensity and efficiency of ongoing natural and human-controlled erosional processes.

## Final highlights

In many regions of the planet, the preservation of anthropogenic landforms and their intrinsic archaeological record is today threatened by ongoing surface processes, and in some cases such processes area accelerated by human agency. In other words, humans built anthropogenic landforms and archaeological sites and today the same force is becoming a menace for the cultural landscape. This is especially true for the arid regions of Western Asia, where ongoing climate change and increase demographic pressure is dramatically increasing the severity of geomorphological processes^70^.

Our geomorphological analysis suggests that, besides natural surface processes, human and animal agency on tell sites are resulting in rapid and negative effects. In fact, anthropogenic disturbances related to cultivation and animals’ impact (burrowing and trampling) alter the stability of the original archaeological stratigraphy, leading to a progressive loss of archaeological heritage.

Our innovative attempt to understand the rate of ongoing surface processes and impacts on tell sites using the RUSLE model is reliable. This automated geomorphological tool developed in the framework of soil loss appears to be efficient also in the context of archaeological tell sites. In fact, our investigation demonstrates the possibility to quantify the risk of losing archaeological soil and/or sediments due to slope erosion and to identify the areas more prone to destruction of anthropogenic landforms and their archaeological record. In this perspective, the procedure based on the RUSLE models tested at Helawa and Aliawa can be applied to a broader number of sites as a predictive geomorphological tool. In this respect, the RUSLE would support archaeologists and conservationists in planning specific archaeological operations aimed at investigating the most threatened parts of mounds’ stratigraphy or to propose specific restoration/preservation strategies to mitigate the risk of loss of the archaeological record. Finally, this low-cost approach can be routinely applied to all the tell sites of the Near East as much as in archaeological contexts worldwide to assess the susceptibility of archaeological heritage to the geomorphological risk promoted by ongoing climate change and promote their conservation and promotion. In fact, the documentation of the preservation of archaeological monuments/sites and the assessment of the potential risk of destruction of cultural heritage at the scale of the single archaeological site are becoming urgent^71^ considering the increasing human pressure on archaeological areas and the acceleration of surface processes pushed by ongoing climate change.

## Supplementary Information

Supplementary Information.

## Data Availability

All data generated or analysed during this study are included in this published article.
