# Supplementary material for: Geomorphological assessment of the preservation of archaeological tell sites
Source: Sci Rep. 2023 May 18;13:7683. doi: 10.1038/s41598-023-34490-4 (PMC10195873; doi:10.1038/s41598-023-34490-4)
Supplement: Supplementary file 1 — Supplementary Information. [file 41598_2023_34490_MOESM1_ESM.docx]

**Supplementary material for:**

**Geomorphological assessment of the preservation of archaeological tell sites**

Luca Forti^1,2^*, Filippo Brandolini^3^, Valentina Oselini^4^, Luca Peyronel^5^, Andrea Pezzotta^1^, Agnese Vacca^5^, Andrea Zerboni^1^

^1)^ Dipartimento di Scienze della Terra “A. Desio”, Università degli Studi di Milano, via L. Mangiagalli 34, 20133 Milano, Italy.

^2)^ Istituto di Geoscienze e Georisorse, Consiglio Nazionale delle Ricerche, Via G. Moruzzi 1, 56124 Pisa, Italy.

^3)^ McCord Centre for Landscape - School of History, Classics and Archaeology Newcastle University, Armstrong Building, Newcastle upon Tyne - NE17RU (UK)

^4)^ Department of Civil, Chemical, Environmental, and Materials Engineering, Alma Mater Studiorum University of Bologna, viale Risorgimento 2, 40136 Bologna, Italy.

^5)^ Dipartimento di Studi Letterari, Filologici e Linguistici, Università degli Studi di Milano, via Festa del Perdono 3, 20122 Milano, Italy.

*Corresponding author's e-mail: [luca.forti@unimi.it](mailto:luca.forti@unimi.it)


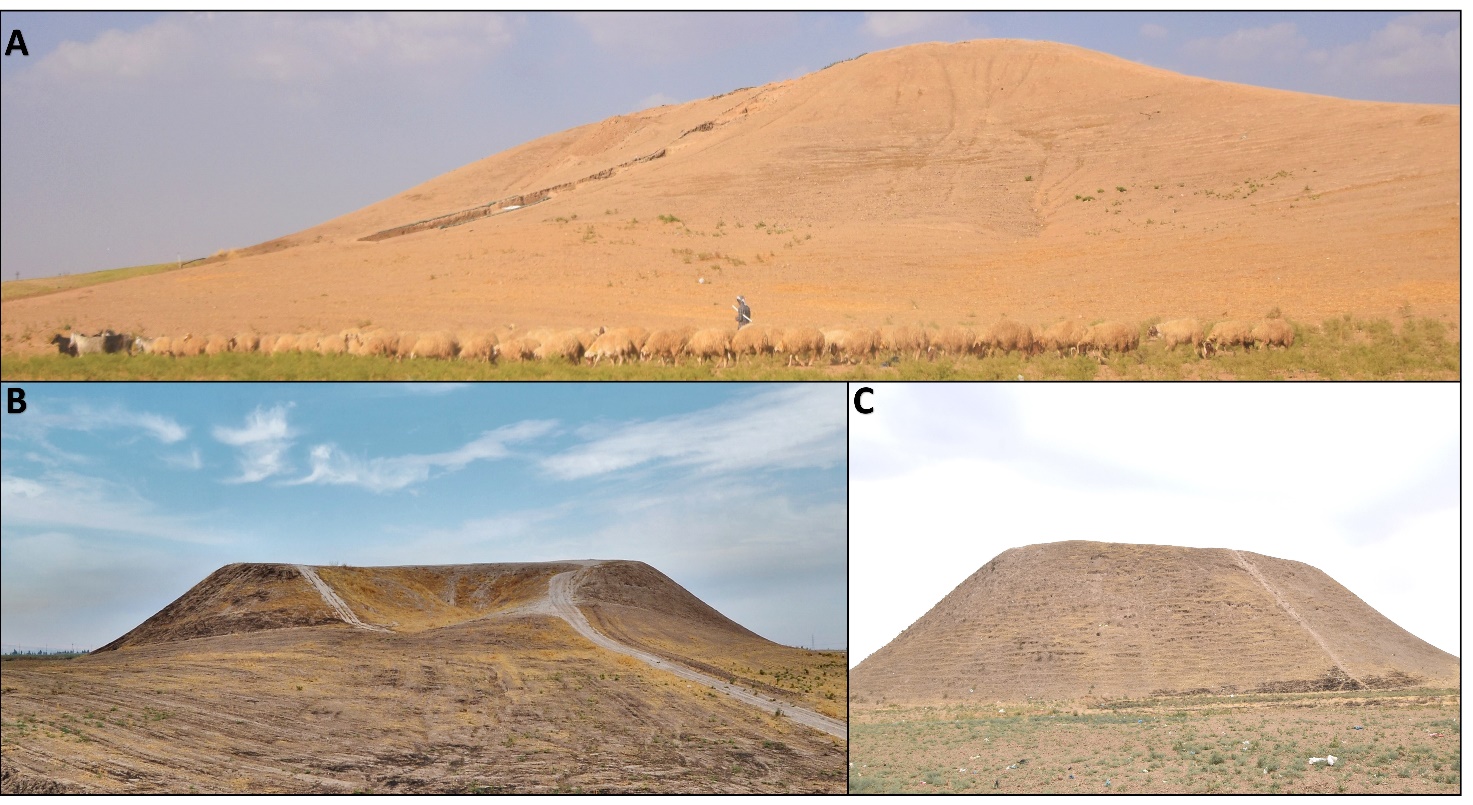


**Figure S1.** (A) Field picture of Tell Helawa (year 2021) illustrating its southern toeslope that is daily exploited for sheep and goat grazing. (B) The gently slope at the southern side of the of Tell Aliawa, showing the central main badland basin. (C) The steep northern slope of Tell Aliawa modelled by the contour track sheep and animal burrows. (Pictures by L. Forti; Archive of the MAIPE Archaeological Project of the University of Milan).
